# Clinically Distinct Phenotypes of Canavan Disease Correlate with Residual Aspartoacylase Enzyme Activity

**DOI:** 10.1002/humu.23181

**Published:** 2017-02-14

**Authors:** Marisa I Mendes, Desirée EC Smith, Ana Pop, Pascal Lennertz, Matilde R Fernandez Ojeda, Warsha A Kanhai, Silvy JM van Dooren, Yair Anikster, Ivo Barić, Caroline Boelen, Jaime Campistol, Lonneke de Boer, Ariana Kariminejad, Hulya Kayserili, Agathe Roubertie, Krijn T Verbruggen, Christine Vianey‐Saban, Monique Williams, Gajja S Salomons

**Affiliations:** ^1^ Department of Clinical ChemistryMetabolic UnitVU University Medical CenterAmsterdam NeuroscienceAmsterdamThe Netherlands; ^2^ Edmond and Lily Safra Children's HospitalSheba Medical Center and Sackler School of MedicineTel Aviv UniversityIsrael; ^3^ Department of PediatricsUniversity Hospital Center Zagreb & University of Zagreb, School of MedicineZagrebCroatia; ^4^ Department of PediatricsAdmiraal De Ruyter ZiekenhuisGoesZeelandThe Netherlands; ^5^ Neurology DepartmentCIBERER ISCIIIHospital Sant Joan de DeuUniversity of BarcelonaBarcelonaSpain; ^6^ Department of pediatrics, metabolic diseasesRadboud University Medical CenterNijmegenThe Netherlands; ^7^ Kariminejad‐Najmabadi Pathology & Genetics CenterTehranIran; ^8^ Medical Genetics DepartmentKoç University School of Medicine (KUSOM)IstanbulTurkey; ^9^ Département de NeuropédiatrieHopital Gui de ChauliacMontpellierLanguedoc‐RoussillonFrance; ^10^ INSERM U1051Institut des Neurosciences de MontpellierMontpellierFrance; ^11^ Beatrix Children's Hospital, University Medical Center GroningenUniversity of GroningenGroningenThe Netherlands; ^12^ Centre de Biologie et de Pathologie Est CHU de LyonService Maladies Héréditaires du Métabolisme et Dépistage NéonatalLyonFrance

**Keywords:** Canavan disease, *ASPA*, ASPA activity, missense variants, clinical phenotype, functional assay

## Abstract

We describe 14 patients with 12 novel missense mutations in *ASPA*, the gene causing Canavan disease (CD). We developed a method to study the effect of these 12 variants on the function of aspartoacylase—the hydrolysis of N‐acetyl‐l‐aspartic acid (NAA) to aspartate and acetate. The wild‐type *ASPA* open reading frame (ORF) and the ORFs containing each of the variants were transfected into HEK293 cells. Enzyme activity was determined by incubating cell lysates with NAA and measuring the released aspartic acid by LC–MS/MS. Clinical data were obtained for 11 patients by means of questionnaires. Four patients presented with a non‐typical clinical picture or with the milder form of CD, whereas seven presented with severe CD. The mutations found in the mild patients corresponded to the variants with the highest residual enzyme activities, suggesting that this assay can help evaluate unknown variants found in patients with atypical presentation. We have detected a correlation between clinical presentation, enzyme activity, and genotype for CD.

## Introduction

Canavan disease (CD, MIM# 271900) is an autosomal recessive neurodegenerative disease characterized by spongy degeneration of the white matter of the brain [Canavan, 1931; van Bogaert and Bertrand, 1949]. It is caused by deficiency of the enzyme aspartoacylase (ASPA) [Matalon et al., 1988]. Symptoms usually appear after normal development during the first months of life and progress rapidly. They include macrocephaly, hypotonia, loss of muscle control, feeding difficulties, developmental delay (including motor and verbal skills), and visual impairment. Some patients develop seizures [Traeger and Rapin, 1998; Surendran et al., 2003b]. At this moment, there is no proven therapy for CD. Use of gene therapy is being investigated and clinical trials are being performed, some of which show limited success in slowing the progression of the disease [Leone et al., 2012]. ASPA is the only known enzyme able to hydrolyze N‐acetyl‐l‐aspartic acid (NAA) into aspartate (Asp) and acetate. ASPA deficiency results in accumulation of NAA in body fluids. Elevation of NAA levels in urine is the biochemical hallmark of CD. Molecular analysis of *ASPA* gene (MIM# 608034, NG_008399.1) is the confirmative test. This gene is located on chromosome 17 (17p13.2). It is composed of six exons and comprises about 30 kb. More than 100 mutations in *ASPA* have been submitted to the Leiden Open Variation Database (LOVD), including deletions, missense mutations, and nonsense mutations (http://www.lovd.nl/ASPA). CD carrier frequency among Ashkenazi Jewish populations is relatively high: one in 30 to one in 60, corresponding to an incidence of one in 5,000 to one in 6,700 [Kronn et al., 1995; Matalon et al., 1995; Feigenbaum et al., 2004]. Only two mutations are responsible for about 98% of CD alleles in this population [Matalon, 1997]. The mutation c.854A>C, p.Glu285Ala is responsible for about 84% of CD alleles, whereas the mutation c.693C>A, p.Y231X is found in about 14% of CD alleles. Both mutations are associated with a severe phenotype. The overall incidence is much lower (one in 200,000 to one to 400,000). Here, the most common mutation responsible for about 40%–48% of the CD alleles is c.914C>A, p.Ala305Glu. This mutation has been associated with both severe and mild phenotypes [Shaag et al., 1995].

ASPA (EC3.5.1.15) is a dimeric metalloenzyme composed by 37 kDa monomers (313 amino acid residues). It is mainly present in brain white matter (mainly oligodendrocytes) and kidney [Baslow et al., 1999; Sommer and Sass, 2012]. The crystal structure of ASPA has been resolved (Bitto et al., 2007; Le Coq et al., 2008). ASPA binds one atom of Zn per monomer [Le Coq et al., 2006] and this metal is necessary for the enzyme reaction. The amino acid residues involved in Zn binding are His21, Glu24, and His116. The catalytic site is composed of residues Arg63, Asn70, Arg71, Tyr164, Arg168, Glu178, and Tyr288. Residues Arg168 and Tyr288 stabilize the binding of NAA to ASPA. It was shown that due to rare codon usage, eukaryotic expression systems need to be used to properly study enzyme variants [Wang and Viola, 2014]. The vast majority of *ASPA* mutations that have been overexpressed in COS or human embryonic kidney (HEK)293 cells present very low enzyme activity (lower than 5% of wild‐type (WT)) [Janson et al., 2006; Sommer and Sass, 2012; Surendran et al., 2003a].

In this article, we report a method for the characterization of missense variants in *ASPA* gene transiently transfected into HEK293 cells. A total of 14 patients (corresponding to 12 missense variants) were included in our study.

## Material and Methods

### Patients, Sample Collection, Mutational Analysis, and Inclusion Criteria

From 1.1.2002 to 31.12.2015 genomic DNA was isolated from blood of patients suspected of CD on basis of clinical and biochemical findings, such as macrocephaly, hypotonia, developmental delay, and increased urinary excretion of NAA. To analyze the coding region of the *ASPA* gene, the six exons and intron boundaries were amplified by PCR. Sequence analysis was performed in both directions with ABI Prism BigDye Terminator Cycle Sequencing Ready Reaction Kits, in an ABI PRISM 3130xl Genetic Analyzer (Applied Biosystems, Foster City, CA). Sequences were compared with the reference sequence from the GenBank (NG_008399.1) using Mutation Surveyor (Softgenetics, State College, PA). Nucleotide numbering of variants reflects cDNA numbering with +1 corresponding to the A of the ATG translation initiation codon in the reference sequence (NM_000049.2). Fourteen patients, harboring missense mutations not described in literature, were selected for this study. Physicians following these patients were invited to fill in a questionnaire covering clinical signs, clinical course, motor and cognitive development, NAA levels, and radiographic investigations. Eleven questionnaires were received. Patients were numbered 1–11 and divided into two groups based on their ability to achieve (minimal) speech and their ability to walk.

### Construction of the ASPA Expression Vector and Site‐Directed Mutagenesis to Introduce Variants

The human *ASPA* gene (NM_000049.2) encoding the enzyme ASPA was amplified by PCR using the primers ASPA_Clon_F, containing a restriction site for the enzyme Asi SI, and ASPA_Clon_R, containing a restriction site of the enzyme Rsr II (Supp. Table S1). After purification, the amplified fragment and the pCMV6 vector (Origene, Rockville, MD) were digested with the enzymes Asi SI (New England Biolabs, Ipswich, MA) and Rsr II (New England Biolabs) and ligated, in frame with mGFP. The final construct was used as a template to introduce the following mutations: c.70G>A (p.Glu24Lys), c.89T>C (p.Leu30Pro), c.170C>T (p.Ala57Val), c.188G>C (p.Arg63Thr), c.206T>G (p.Leu69Arg), c.302G>T (p.Gly101Val), c.385G>A (p.Glu129Lys), c.509T>C (p.Ile170Thr), c.539G>T (p.Gly180Val), c.610G>C (p.Asp204His), c.743A>G (p.Gln248Arg), c.854A>C (p.Glu285Ala), c.857C>A (p.Ala286Asp), and c.914C>A (p.Ala305Glu) by site‐directed mutagenesis using the primers listed in Supp. Table 1, as described previously [Betsalel et al., 2012]. The authenticity of mutagenesis and cloning was verified by DNA sequencing.

### Overexpression of ASPA Alleles in HEK293 Cells

HEK293 cells were cultured to 70% confluency and used for transient expression of the obtained constructs. For the transfections, 12 μg of plasmid DNA was incubated with 60 μl of Fugene (Promega, Madison, WI) at room temperature for 15 min in serum‐free DMEM medium (Thermo Fisher Scientific, Waltham, MA). The complex was applied to the cells. Cells transfected either with WT pCMV6‐*ASPA* (from here on related to as WT), the empty vector (mock transfected) as well as untransfected cells were included as controls. The most common variants found in patients from Ashkenazi Jewish background (c.854A>C, p.Glu285Ala) and in non‐Jewish populations (c.914C>A, p.Ala305Glu) were also included as controls. Fluorescent microscopy was used to estimate transfection efficiency. Cells were harvested 48 hr after transfection, flash‐frozen, and stored at −80°C until further use. Transfections were performed in triplicate.

### Functional Analysis of ASPA Variants by Novel Enzyme Assay

A novel enzyme assay was developed in which the production of Asp from NAA was measured by LC–MS/MS. Cell pellets were suspended in 200 μl of 200 mmol/l Tris buffer (pH = 8.5) containing 0.5 mmol/l phenylmethylsulfonyl fluoride. Cell membranes were disrupted by three freeze–thaw cycles. The supernatant was collected following 5 min centrifugation at 13,000*g* (11,000 rpm) at 4°C. Approximately 10 μg of protein (1 μg for WT) was incubated for 30 min at 37°C in 200 μl of 200 mmol/l Tris buffer (pH = 8.5) containing 1.25 mmol/l of NAA. The reaction was terminated by placing the samples on ice and adding 20 μl 200 mmol/l nonafluoropentanoic acid. After 25 μl of [D3]‐aspartic acid was added as an internal standard, the lysates were pipetted on a molecular weight cut‐off filter (mw 10.000; Amicon; Millipore, Billerica, MA) and centrifuged for 10 min at 20,000*g* (14,000 rpm) at 4°C. *K*_m_ value for WT was found to be 0.2 mmol/l, in agreement with previously published data [Le Coq J. et al., 2006].

The aspartic acid formed was measured by positive electrospray triple quadrupole tandem mass spectrometry (API 5000; Applied Biosystems) coupled to a Shimadzu Nexera HPLC (Kyoto, Japan). Five microliters of the incubation mixture was separated on a Symmetry Shield C18 analytical column (3.5 μm, 3.0 × 100 mm^2^; Waters, Milford, MA) using a mobile phase containing 5 mmol/l nonafluoropentanoic acid. The gradient was started at 0% acetonitrile for 5 min. In 4 min, the acetonitrile content of the mobile phase was increased from 0% to 50%. Mass transitions *m*/*z* 134.1 → 88.1 and 137.1 → 91.1 were used for unlabeled and [D3] labeled aspartic acid. Intra‐assay variations were 11% for WT and 4% for a variant with low activity (*n* = 6). Protein content was measured using the Bicinchoninic acid method (Sigma–Aldrich, St Louis, MI). Bovine serum albumin was used as the standard. All determinations were performed in triplicate. The amounts of aspartic acid measured in WT and variant ASPA enzymes were corrected for endogenous aspartic acid and endogenous ASPA activity by subtracting the amount of aspartic acid measured in mock‐transfected cells.

### Western Blot

To confirm the presence of ASPA protein in the transfected cells, a Western blot was performed. Cell pellets were resuspended in urea lysis buffer (10 mmol/l Tris HCl, 8 mol/l urea, 100 mmol/l NaCl, pH 8.0). After DNA shearing using a 29 gauge needle, protein concentration was determined as described above and 30 μg of total protein was separated in a 12% stain‐free SDS gel (Bio‐Rad Laboratories, Hercules, CA). Proteins were transferred onto a polyvinylidenfluoride membrane (Bio‐Rad) using a Trans‐Blot Turbo Transfer System (Bio‐Rad). Immunodetection was performed using a primary antibody (rabbit) directed at the GFP tag (ab290; Abcam, Cambridge, UK) and a secondary anti‐rabbit antibody (PO448; Dako, Glostrup, Denmark). Immune complexes were detected by enhanced chemiluminescence (Lumilight Plus), according to the manufacturer's specifications (Roche, Indianapolis, IN). Images were acquired in a CCD (charge‐coupled device) imager ChemiDoc XRS (Bio‐Rad) using the Image Lab software (Bio‐Rad).

### Structural Modeling

The crystal structure of the WT human ASPA (PDB ID:2O4H) was used as a template to generate structures for the variants addressed in this study using the online structural predictor SDM (http://mordred.bioc.cam.ac.uk/~sdm/sdm.php). Molecular graphics and analyses were performed with the UCSF Chimera package. Chimera is developed by the Resource for Biocomputing, Visualization, and Informatics at the University of California, San Francisco (supported by NIGMS P41‐GM103311) [Pettersen et al., 2004].

## Results

### Genotypic Characterization of Patients

**Figure 1:**
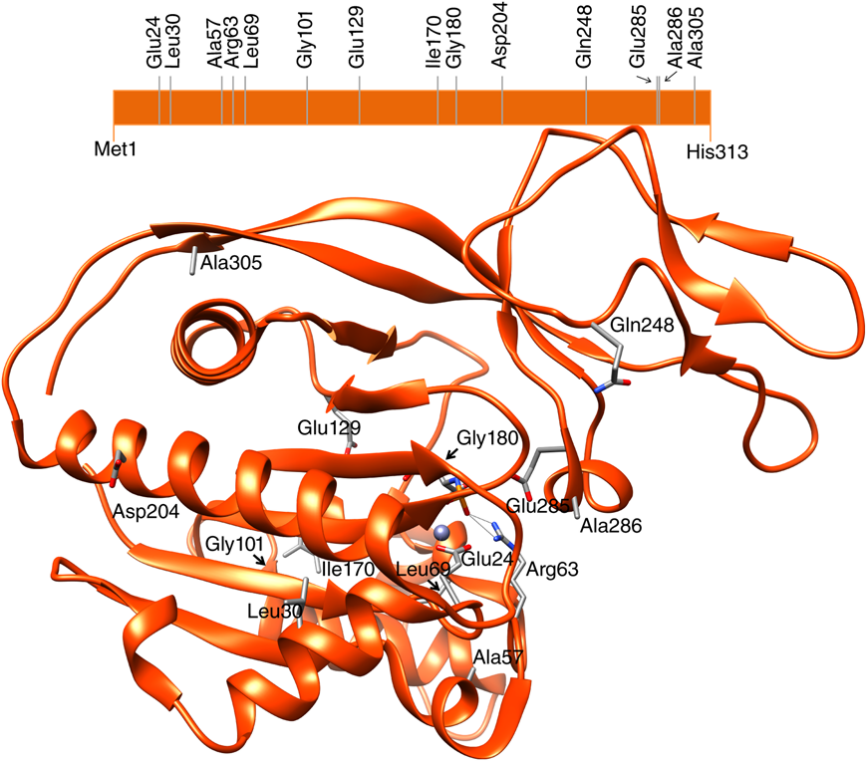
Schematic and structural representation of the ASPA monomer with the location of the amino acid residues addressed in this study. The structural image was generated using the crystal structure of ASPA (PDB ID: 2O4H), obtained using the UCSF Chimera package. Chimera is developed by the Resource for Biocomputing, Visualization, and Informatics at the University of California, San Francisco (supported by NIGMS P41‐GM103311).

**Figure 2:**
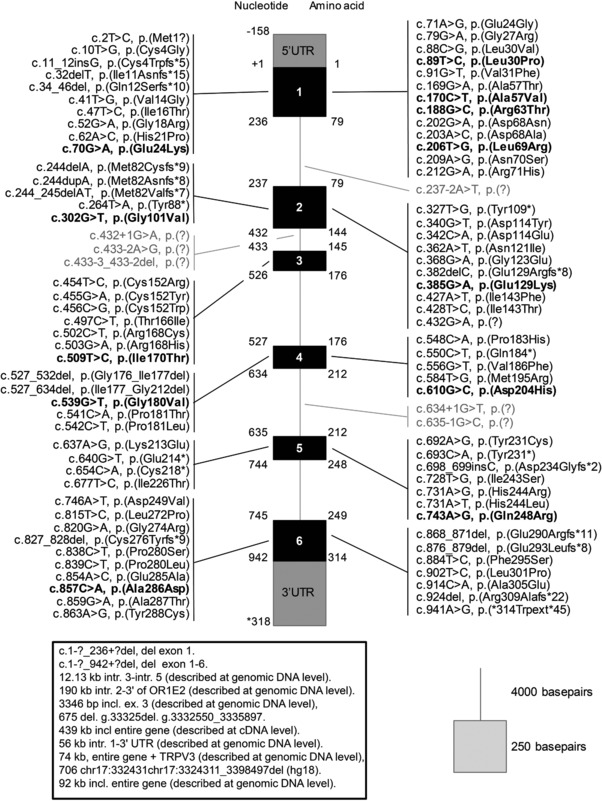
Schematic representation of the *ASPA* gene and the distribution of all published mutations (as of September 2016). These mutations are part of the mutation database LOVD (http://www.lovd.nl/ASPA). Variants located in exonic regions are in black, variants in intronic regions are in gray. Novel variants addressed in this study are represented in bold. Large deletions are depicted in the box.

DNA sequencing was used to confirm diagnosis of CD in 84 patients in our laboratory. Twelve novel missense variants were detected in 14 patients, who were selected for this study: nine homozygous and five compound heterozygous patients. Figure 1 depicts the structure of the ASPA monomer with the location of the 12 affected residues as well as the two most common variants. All mutations result in the replacement of amino acid residues that are very conserved across species. The identified mutations have been included in the Leiden LOVD (http://www.lovd.nl/ASPA). A scheme of all mutations reported in *ASPA* gene is depicted in Figure 2.

### Clinical Features of Patients with New ASPA Mutations

Eleven out of the 14 questionnaires sent out to the clinicians were returned. In one case, the clinician could not be contacted, the other two did not respond. Eleven out of 14 Canavan patients (six males) with new missense mutations were evaluated. First signs in this patient group appeared from birth (macrocephaly) up to an age of 6 years and 10 months. Follow‐up time was 6 months to 36 years (range 6–432 months, average 153, median 120 months). Four patients were lost to follow up (at 6, 24, 264, and 432 months); two of these had died. At the last follow up, six patients were still alive; of one this is unknown. First signs (provided for nine out of 11 patients) included developmental delay, hypotonia (head lag (*n* = 4), poor/no head control (*n* = 4)), decreased contact (*n* = 2), motor retardation (*n* = 1), movement abnormalities (*n* = 1), epilepsy (*n* = 1), intellectual disability (*n* = 1), loosing skills (*n* = 1), and behavioral problems (*n* = 1). Patients were categorized into two groups based on clinical presentation. The atypical/mild group was composed of patients 1–4. Patients 5–11 were part of the severely affected group.

Clinical course in patients was unknown (*n* = 1), stable (*n* = 2), slowly progressive (*n* = 8), or rapidly progressive (*n* = 1). Rapid deterioration was described in four patients and was determined by the development of seizures in patients with either a slowly progressive (patients 2, 5, and 8) or rapidly progressive disease course (patient 9). Other signs during the course of disease were optic atrophy (one out of seven), developing spastic tetraparesis (three out of six), dysarthria (two out of 10) and nystagmus (one out of 10), amblyopia (one out of 10) and intermittent exotropia alternans (one out of 10).

Head circumference was normal in patients 1, 2, 3, and 5. Macrocephaly was seen in all other patients with a severe phenotype; patient 10 was normocephalic at birth and developed macrocephaly with time. In nine out of 11 patients, height was within the normal range (–2SD to +2SD). Seven patients had a normal weight, whereas one was overweight (patient 3). No information on weight was available for two patients.

Seizures were present in four out of 11 patients, starting from an age of 3 months to 8 years. Seizures were treated with either valproate or phenobarbital and topiramate (no information in one). Two patients (number 5 and 10) received uncommon drugs (lithium citrate or acetazolamide) without effect.

Cognition was affected in all patients. Cognition was reported as severely deficient in seven patients. Five patients showed no speech development at all. In patients 1–4 (mild types), speech was less severely affected, with two of these patients receiving special or supportive education, the latter attending normal school (no information from patient 3). Of the severely affected group, patient 10 received special education with mild deficient cognition. In all other patients, no education was possible.

All except four patients showed abnormal motor development. Patients 1, 2, and 3 were able to walk without support. Patient 4 walked with support until the age of 13 years and became wheelchair dependent. All patients classified as severe (patients 5–11) showed a severe motor impairment and did not achieve walking. These patients also suffered from poor to no head control.

EEGs were performed in eight out of 11 patients. In five patients, EEGs were diffusely slow; in one patient, paroxysmal features were observed, with a normal background activity, and in another, diffuse dysrhythmic changes (patient 2). In one patient, clinical seizures were observed but could not be confirmed on EEG. No EEGs were performed in the other three patients because they did not show clinical evidence of seizures.

An MRI was performed in 10 out of 11 patients. In four patients, an MRS (patients 1, 9, 10, and 11) was included in the same investigation. Patients categorized as mild or atypical presented MRI/MRS not typical for classical Canavan's disease, in agreement with their clinical presentations.

A summary of genotype and phenotypic presentation of disease for all patients is described in Table 1.

**Table 1:** Genotypic, Clinical, and Biochemical Data of Patients with Novel *ASPA* Mutations*

Patient number	1	2	3	4	5	6	7	8^a^	9^a^	10	11
Genotype^b^	**c.509T>C, p.Ile170Thr** c.914C>A, p.Ala305Glu	**c.302G>T, p.Gly101val** c.432G>A, p.(?)	**c.509T>C, p.Ile170Thr**	**c.610G>C, p.Asp204His**	**c.743A>G, p.Gln248Arg**	**c.89T>C, p.Leu30Pro**	**c.170C>T, p.Ala57Val** c.914C>A, p.Ala305Glu	**c.70G>A, p.Glu24Lys**	**c.70G>A, p.Glu24Lys**	**c.385G>A, p.Glu129Lys** c.503G>A, p.Arg168His	**c.539G>T, p.Gly180Val** c.556G>T, p.Val186Phe
Presentation (months)	48	54^c^	19	3	4	5	1	6	3	2	6
Macrocephaly	–	–	–	+	–	+	+	+	+	+	+
Epilepsy	–	–	–	+	+	–	–	–	+	+	–
Mental delay	+	+^d^	++^e^	+++	+++	+++	+++	+++	+++	+	+++
No speech	–	–	–	–	+	+	+	+	+	+	?^f^
Maximum speech	Normal language	Normal language	Words	Short sentences	Sounds	Sounds	Sounds	Sounds	Sounds	No	?^f^
Motor delay	–	+	–	+	+++	+++	+++	+++	+++	+++	++
Poor head control	–	–	–	+	+	+	+	+	+	+	+
No walking achieved	–	–	–	–	+	+	+	+	+	+	?^f^
Increased NAA excretion (urine)	+	+	+	+	+	+	+	+	‐	+	+
Follow up duration (months)	134	432^g^	120	372	109	24^g^	264^g^	15	92	120	6^g^
Enzyme activity (% of WT) in HEK293 transfectants^h^	5.5 ± 2.8	<1%	5.5 ± 2.8	12.4 ± 2.8	<1%	<1%	<1%	<1%	<1%	<1%	<1%

^*^ of whom we received questionnaires. Clinical data from homozygous patients 12 (**c.188G>C, p.Arg63Thr**), 13 (**c.206T>G, p.Leu69Arg**), and 14 (**c.857C>A, p.Ala286Asp**) are not available.

*Notes*: A + indicates the presence and a – indicates the absence of the phenotype. For mental and motor delay + means mild, ++ is moderate, and +++ means severe.

a Patients are siblings.

b One variant is shown for homozygous patients. Novel variants are highlighted in bold.

c Infancy.

d Attended normal school with support.

e Autism.

f Did not achieve normal milestone age.

g Lost to follow up at.

h For heterozygous patients, the activity indicated corresponds to the variant in bold.

### Functional Analysis of Novel ASPA Variants

Enzyme activity was determined in cell lysates of HEK293 transfected with the constructs described. Cell lysates transfected with WT *ASPA* displayed an activity of 6,180 ± 1,223 nmol Asp/h/mg. This activity was 200‐fold higher than the mock transfected lysates. Activities are expressed as % of WT activity. The majority of the studied variants presented with enzyme activities lower than 1% of the WT. The common variant p.Glu285Ala presented an activity of 2.7 ± 0.7% of the WT activity. The variant p.Ile170Thr presented 5.5 ± 2.8% of residual activity. The variant with the highest activity detected (12.4 ± 2.8%) was p.Asp204His.

### Western Blot

**Figure 3:**
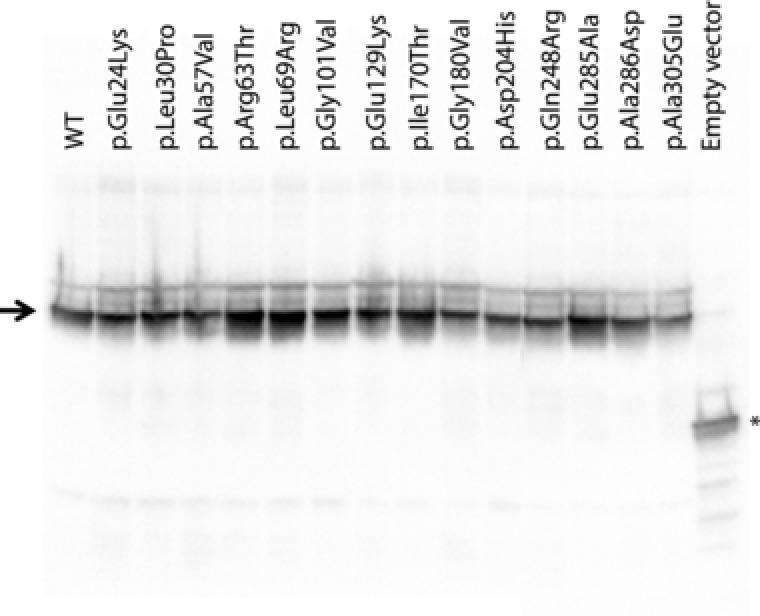
Expression levels of ASPA proteins in fusion with mGFP transiently transfected into Human Embryonic Kidney cells (HEK293) monitored by Western blot analysis after SDS‐PAGE. Thirty micrograms of total protein was loaded per lane. An antibody against mGFP was used as primary antibody. The arrow points to the ASPA‐mGFP fusion protein. The ^*^ indicates free mGFP.

Since most of the variants studied presented with very low enzyme activities, a Western Blot was performed to verify whether all variant proteins were expressed. All variants presented detectable expression of ASPA with no major differences concerning level of expression when compared to the WT (Fig. 3).

### Structural Analysis

Structural models for the 12 novel variants studied were generated (using the prediction tool SDM as described in section *Materials and Methods*) and compared with the WT structure. Overall the amino acid replacements did not cause major structural changes. This was also observed when Wijayasinghe et al. (2014) crystalized four ASPA variants. Regarding variant p.Glu24Lys, amino acid residue Glu24 is one of the Zn binding residues. Replacement of the residue Glu by Lys will change the local polarity of the residue and probably interfere with the bond stability of the cofactor, which could explain the decrease in enzyme activity. In addition, variant p.Glu24Ala resulted in complete ablation of enzyme activity [Le Coq et al., 2008]. Replacement by Asp leads to decreased levels of bound Zn and no enzyme activity [Le Coq et al., 2008]. The variant p.Glu24Gly has been reported in heterozygous state in one patient (the other allele harboring c.514C>A, p.Pro181Thr) with classical CD [Zeng et al., 2002].

Regarding variant p.Leu30Pro, the amino acid Leu presents the highest propensity to form α‐helix structures and provide helix stability. Indeed, Leu30 is located in α‐helix 1. On the contrary, Pro cannot form α‐helices. It can only be incorporated in the first turn of the helix due to the ring structure, which is a helix breaker. This amino acid replacement is likely to cause a structural change in ASPA, which can explain the absence of enzyme activity. It was shown before that replacement of Leu by Pro leads to unfolding of helical regions [O'Neil and DeGrado, 1990; Strehlow et al., 1991].

The variant residue of p.Ala57Val, Ala57 is located on the first turn of α‐helix 2. Replacement by Val will not dramatically change the properties of the residue. However, Val residues are most often found in β‐sheet structures and replacement of Ala by Val results most likely in structural changes that can explain the absence of enzyme activity observed for the p.Ala57Val variant.

Regarding variant p.Arg63Thr, amino acid residue Arg63 is part of the catalytic center of ASPA and is important for stabilization of the substrate, meaning that replacement of that residue will likely result in an inactive enzyme, as observed.

In the variant p.Leu69Arg, replacement of the branched non‐polar amino acid residue Leu by the polar amino acid Arg with a longer side chain will likely have impact on the overall structure of the protein and result in a decreased enzyme activity.

The affected residue in variant p.Gly101Val, Gly101 is very conserved in eukaryotes. Val is an amino acid residue with a larger side chain, which can result in a steric hindrance. In our system, this variant presented with no enzyme activity.

Concerning the variant p.Glu129Lys, residue Glu129 contributes to intra‐monomer stability by establishing hydrogen bonds with Thr166. Replacement by Lys will hinder the formation of these stabilizing bonds, which can explain the observed inactivation.

The variant p.Ile170Thr affects residue Ile 170, which is part of α‐helix 7. The in silico model predicts that the replacement of Ile170 by Thr results in disruption of the α‐helix. Although this replacement results in a decrease in enzyme activity (about 6% of WT), it is important to consider that among the studied variants this one presented with the second highest residual enzyme activity.

About variant p.Gly180Val, replacement by Val will likely have an impact on protein structure due to the addition of the side chain.

Variant p.Asp204His affects the very conserved (eukaryotes) Asp204. This residue is located at the surface of the protein. This variant was used for in silico secondary structure prediction and reported to cause conformational changes in the protein [Shinar et al., 2004].

Regarding variant p.Gln248Arg, Gln 248 is located in the dimer interface. Arg presents completely different properties, changing the size and polarity of the side chain of this residue. This replacement may affect the formation of the dimer, which can explain the inactivation of ASPA.

The variant p.Ala286Asp affects a highly conserved amino acid residue in eukaryotes. Ala presents with a small non polar side chain. Replacement by Asp results in a dramatic increase in the size of the side chain. This could explain the non‐detectable enzyme activity.

## Discussion

Diagnosis of inborn errors of metabolism (IEM) has come a long way. For highly incapacitating diseases, like CD, with no available therapy the option of prenatal diagnosis is often very important for families with affected individuals. To prevent false positive or negative results, it is of utmost importance that the method used to diagnose the disorder is both accurate and specific. For CD, historically prenatal diagnosis was based on enzyme determinations in amniocytes. The extremely low ASPA activity in these cells represents an analytical challenge and unfortunately led to misdiagnosed cases in the past [Matalon et al., 1992]. The introduction of stable isotope dilution analysis of amniotic fluid in our laboratory in 1991 [Jakobs et al., 1991] offered a more reliable technique for this purpose. Later DNA analysis replaced these methods for the majority of prenatal diagnoses. Nowadays, next‐generation sequencing is becoming more and more a first step in the diagnosis of patients with disorders of unknown etiology including so far undetected IEM. This results in the identification of novel variants and mild or atypical phenotypic presentations. Therefore, a functional test is necessary to understand the significance of novel missense variants. We developed an overexpression system to characterize missense variants in the *ASPA* gene. This system bypasses the need for additional material from patients and can also be used to confirm pathogenicity of variants identified in familial cases.

More than 100 mutations have been described in the *ASPA* gene. We developed a public database for these variants and included all published and novel variants (http://www.lovd.nl/ASPA). We analyzed the effects of 12 novel *ASPA* missense mutations (detected in a total of 14 patients) as well as two common missense mutations (p.Glu285Ala and p.Ala305Glu). The 14 variants were transiently transfected into HEK293 cells, which are considered the most suitable system to study ASPA variants because it ensures the availability of all necessary codons [Wang and Viola, 2014].

The activity measured for the WT (6,180 ± 1,223 nmol Asp/hr/mg) and for the variant p.Glu285Ala (about 2% of WT) match the activities described in the literature [Kaul et al., 1994; Moore et al., 2003; Sommer and Sass, 2012], validating our method.

Different phenotypic presentations have been described for CD. The classical severe form is the most common, but milder CD presentations have also been reported [Toft et al., 1993; Shaag et al., 1995; Zafeiriou et al., 1999; Tacke et al., 2005; Yalcinkaya et al., 2005; Janson et al., 2006; Velinov et al., 2008; Delaney et al., 2015; Nguyen and Ishak, 2015; Sarret et al., 2016]. In our study, four patients showed an atypical or mild presentation (patients 1–4), whereas the remaining seven presented with the classical CD phenotype. In our study, we observed a correlation between the mutations detected in the mild cases and the variants with the relatively higher enzyme activities (p.Ile170Thr, p.Asp204His).

Based on the functional studies, the biochemistry and the clinical information, we classify the following 10 variants as pathogenic mutations: p.Glu24Lys, p.Leu30Pro, p.Ala57Val, p.Arg63Thr, p.Leu69Arg, p.Gly101Val, p.Glu129Lys, p.Gly180Val, p.Gln248Arg, and p.Ala286Asp. All these variants were identified in patients with a severe phenotypic presentation of CD and showed less than 1% of WT ASPA residual activity in our system. The homozygous p.Asp204His mutation was detected in a 31‐year‐old patient (patient 4). This variant showed a relatively high residual ASPA activity (12%) in our functional assay. The clinical features did fit with mild CD (macrocephaly, MRI characteristic for CD) and also largely increased NAA levels in urine were detected. We believe that variant p.Asp204His is responsible for milder CD presentation and therefore we also classify this variant as pathogenic. The variant p.Ile170Thr is more difficult to classify. The p.Ile170Thr was found in two patients with only a very mild increase of NAA in urine without further hallmark signs of CD. The two patients harboring this variant are currently 10 (patient 3) and 11 years old (patient 1). Because in the two other mild patients (patient 2 and 4), the clinical presentation became more severe with age (around 30 (patient 2) and 14 (patient 4)), the clinical phenotype may change with time. For the time being, we propose to consider this as a rare variant of unknown clinical significance. Identification of more patients and the additional data from disease progression of these two cases will help to assess the clinical significance of this variant. The mild presentation observed for patient 2 could potentially be explained by variant c.432G>A. This variant was described before [Schober et al., 2011]. Nucleotide c.432 is the last base (3′end) of exon 2 and it will most likely affect the splice donor of intron 2, probably resulting in skipping of exon 2 and possibly allowing a small percentage of correctly transcribed *ASPA* mRNAs. The mild phenotype observed in this patient could be explained by the correct WT splicing of a small amount of *ASPA* mRNA product as described for other splice mutations [van Berge et al., 2012] but mRNA studies are necessary to confirm this hypothesis.

We report a correlation between *ASPA* mutation, enzyme activity and clinical presentation of CD. Such a correlation was observed before for CD [Zano et al., 2013]. We describe 14 new patients with a total of 12 novel missense variants not characterized before. By the use of our functional assay, we have confirmed for 11 out of 12 that the variants are pathogenic. Three of the patients that presented with milder phenotype harbored the mutations that presented with the highest residual activities in our assay. It is remarkable that a residual activity of about 10% of WT can already be associated with a milder form of CD. This observation suggests that a slight increase in activity can already have a big impact on the quality of life of patients and families that have to deal with CD. This newly developed functional assay is implemented in our diagnostic facility and will improve not only diagnostics for new cases, but it can also be helpful to identify potential therapies.

*Disclosure statement*: The authors declare no conflict of interests.

## Supporting information

Table S1. Oligonucleotides used for cloning and site directed mutagenesis reactions
