# Supplementary material for: Clinically Distinct Phenotypes of Canavan Disease Correlate with Residual Aspartoacylase Enzyme Activity
Source: Hum Mutat. 2017 Feb 14;38(5):524–31. doi: 10.1002/humu.23181 (PMC5412892; doi:10.1002/humu.23181)
Supplement: Supplementary file 1 — Table S1. Oligonucleotides used for cloning and site directed mutagenesis reactions [file HUMU-38-524-s001.pdf]

Table S1: Oligonucleotides used for cloning and site directed mutagenesis reactions

Primer	Sequence (5' → 3')
ASPA_Clon_F	GAGGCGATCGCCATGACTTCTTGTCACATTGCTGA
ASPA_Clon_R	GCGTCGGTCCGCTCTAATGTAAACAGCAGCGAATA
ASPA_SDM_70G_A_F	CTTTGGAGGAACCCATGGGAATAAGCTAACCGGAG
ASPA_SDM_70G_A_R	CTCCGGTTAGCTTATTCCCATGGGTTCCTCCAAAG
ASPA_SDM_89T_C_F	GCTAACCGGAGTATTTCCGGTTAAGCATTGGCTAG
ASPA_SDM_89T_C_R	CTAGCCAATGCTTAACCGGAAATACTCCGGTTAGC
ASPA_SDM_170C_T_F	CATTTATTACTAACCCCAGAGTAGTGAAGAAGTGTACCAGATA
ASPA_SDM_170C_T_R	TATCTGGTACACTTCTTCACTACTCTGGGGTTAGTAATAAATG
ASPA_SDM_188G_C_F	GCAGTGAAGAAGTGTACCACATATATTGACTGTGACCTG
ASPA_SDM_188G_C_R	CAGGTCACAGTCAATATATGTGGTACACTTCTTCACTGC
ASPA_SDM_206T_G_F	CAGATATATTGACTGTGACCGGAATCGCATTTTTGACCTTG
ASPA_SDM_206T_G_R	CAAGGTCAAAAATGCGATTCCGGTCACAGTCAATATATCTG
ASPA_SDM_302G_T_F	AGGGCTCAAGAAATAAATCATTTATTTGTTCCAAAAGACAGTGAAGAT
ASPA_SDM_302G_T_R	ATCTTCACTGTCTTTTGGAACAAATAAATGATTTATTTCTTGAGCCCT
ASPA_SDM_385G_A_F	CATGGGGTGCACTCTTATTCTTAAGGATTCCAGGAATA
ASPA_SDM_385G_A_R	TATTCCTGGAATCCTTAAGAATAAGAGTGCACCCCATG
ASPA_SDM_509T_C_F	TATGCGACCACTCGTTCCACAGCCAAGTATCCTG
ASPA_SDM_509T_C_R	CAGGATACTTGGCTGTGGAACGAGTGGTCGCATA
ASPA_SDM_539G_T_F	TGTGGGTATAGAAGTTGTTCCTCAGCCTCAAGGG
ASPA_SDM_539G_T_R	CCCTTGAGGCTGAGGAACAACTTCTATACCCACA
ASPA_SDM_610G_C_F	AAATGAGAAAAATGATTAAACATGCTCTTCATTTTATACATCATTTCAATGAAGGAAAA
ASPA_SDM_610G_C_R	TTTTCCTTCATTGAAATGATGTATAAAATGAAGAGCATGTTTAATCATTTTTCTCATTT
ASPA_SDM_743A_G_F	CATCCATCCTAATCTGCGGGATCAAGACTGGAAAC
ASPA_SDM_743A_G_R	GTTTCCAGTCTTGATCCCGCAGATTAGGATGGATG
ASPA_SDM_854A_C_F	GTACCCCGTGTTTGTGAATGCGGACGCATATTACGAAAAG
ASPA_SDM_854A_C_R	CTTTTCGTAATATGCGTCCGCATTCACAAACACGGGGTAC
ASPA_SDM_857C_A_F	CCCGTGTTTGTGAATGAGGACGCATATTACGAAAAGAAA
ASPA_SDM_857C_A_R	TTTCTTTTCGTAATATGCGTCCTCATTCACAAACACGGG
ASPA_SDM_914C_A_F	GACAACTAAACTAACGCTCAATGAAAAAAGTATTCGCTGCTGTTTAC
ASPA_SDM_914C_A_R	GTAAACAGCAGCGAATACTTTTTTCATTGAGCGTTAGTTTAGTTGTC

Note: F – Forward, R – Reverse. Mismatch nucleotides are underlined.
